# Is the Mediterranean Diet Pattern Associated with Weight Related Health Complications in Adults? A Cross-Sectional Study of Australian Health Survey

**DOI:** 10.3390/nu13113905

**Published:** 2021-10-30

**Authors:** Canaan Negash Seifu, Paul Patrick Fahey, Kedir Yimam Ahmed, Evan Atlantis

**Affiliations:** 1 School of Health Sciences, Western Sydney University, Penrith, NSW 2751, Australia; c.seifu@westernsydney.edu.au (C.N.S.); p.fahey@westernsydney.edu.au (P.P.F.); 2 Translational Health Research Institute, Western Sydney University, Penrith, NSW 2751, Australia; k.ahmed@westernsydney.edu.au; 3 Discipline of Medicine, Faculty of Medicine and Health, Nepean Clinical School, The University of Sydney, Sydney, NSW 2006, Australia

**Keywords:** mediterranean diet, unhealthy diet, weight related complications, health conditions, obesity, Edmonton Obesity Staging System

## Abstract

We hypothesized that unhealthy dietary pattern would be associated with weight related complications among overweight. We analysed data from the Australian Health Survey conducted from 2011 to 2013. A total of 5055 adults with at least overweight (body mass index ≥25 kg/m^2^) were analysed. We used logistic regression to assess the association between unhealthy dietary pattern, defined by low adherence to Mediterranean Diet Score (MDS), and weight related complications, defined by the Edmonton Obesity Staging System (EOSS). We repeated the logistic regression models by age and socio-economic disadvantage strata in sensitivity analyses. We also repeated the main analysis on a propensity score matched dataset (*n* = 3364). Complications by EOSS ≥2 was present in 3036 (60.1%) participants. There was no statistically significant association between unhealthy dietary pattern and weight related complication (odds ratio 0.98 (95%confidence interval: 0.85, 1.12)). The null association remained the same after repeating the analysis on three age and five socio-economic indexes for areas strata. The finding persisted after the analysis was repeated on a propensity score matched dataset. We found no evidence to support the hypothesis that unhealthy dietary pattern was associated with weight related complications in this cross-sectional study of the Australian population with overweight or obesity.

## 1. Introduction

Overweight and obesity is a major global health issue. It is estimated that more than 1.9 billion adults (aged 18 years and over) worldwide had overweight and obesity in 2016 [1]. Excess body weight, defined by Body Mass Index (BMI) of at least 25, is one of the leading factors believed to be causally associated with increased fatal and non-fatal disease outcomes globally [2]. For instance, there is an elevated risk for non-communicable disease risk such as type 2 diabetes, coronary heart disease, stroke, asthma, and several cancers [3,4,5]. Studies have shown that metabolically healthy obesity, especially when combined with a high level of fitness, is associated with lower disease risk [6]. However, longitudinal studies have showed that metabolically healthy individuals living with obesity can develop weight related complications [7,8].

The Edmonton Obesity Staging System (EOSS) [9] has become one of the most widely researched clinical obesity staging systems after it was introduced in 2009 [10]. It is a five-stage system that categorizes individuals with overweight and obesity according to the severity of weight related complications. The EOSS has been shown to be useful for predicting risk and benefits of surgical and non-surgical weight management treatments in clinical populations. It also differentiates mortality, health service use (including polypharmacy), poor work, and mortality outcomes [11]. Therefore, a better understanding of risk factors associated with weight related complications defined using the EOSS could help with the development of new public health prevention strategies.

Unhealthy dietary pattern may be a useful indicator of weight related complications, as it has been consistently associated with an increased prevalence of overweight and obesity in population-wide studies [12,13]. Further, evidence from a recently published umbrella review suggests that adherence to a healthy dietary pattern such as the Mediterranean diet is associated with a decreased risk of weight gain or obesity [14]. The Mediterranean diet pattern has been linked with health benefits [15] and is considered a healthy dietary pattern by dietary guidelines [16]. Only a few studies with inconsistent findings have investigated associations between diet/dietary patterns and weight related complications [17,18]. Therefore, we aimed to explore the association between an unhealthy dietary pattern (defined by low adherence to the Mediterranean diet pattern) and weight related complications (defined by the EOSS ≥ 2) in Australian adults with overweight or obesity.

## 2. Materials and Methods

We present this study according to the journal’s formatting requirements and the STROBE guideline for reporting cross-sectional studies [19].

### 2.1. Study Design, Setting and Participants

The Australian Bureau of Statistics (ABS) conducted the Australian Health Survey (AHS) in 2011–2013 that contained the National Nutrition and Physical Activity Survey (NNPAS), the National Health Survey (NHS), and the National Health Measures Survey (NHMS). Study participants who were in the NHS or NNPAS and gave consent to the NHMS provided urine and blood samples; the samples were then tested for a range of chronic disease biomarkers. We used data from the NNPAS and NHMS, with 9435 fully responding adults. For the present study, we selected participants who were adults (aged 18 years or over) with at least overweight (BMI ≥ 25 kg/m^2^) and not pregnant or breast feeding at the time of the survey (*n* = 5055). Since a comprehensive description of the methodology used in this study has already been published [20], we present a brief summary below. 

### 2.2. Ethics 

The AHS was approved by all relevant ethics committee(s) and was conducted according to Australian law (Census and Statistics Act 1905) [21] and the principles expressed in the Declaration of Helsinki. Also, a written informed consent was sought from study participants [20].

### 2.3. Data Sources/Measurement 

The NNPAS was conducted from May 2011 to June 2012 [20]. Trained ABS interviewers used a Computer Assisted Personal Interview (CAPI) methodology to collect self-reported data. Dietary data was collected using the 24-h recall methodology. The interviewers used the Automated Multiple-Pass Method (AMPM) developed by the Agricultural Research Service of the United States Department of Agriculture [22]. It was adapted by the ABS and the Food Standards Australia New Zealand to reflect the Australian food supply. The AMPM has five phases: quick list, forgotten foods, time and occasion, detail cycle, and final probe that encourage participants to think about foods and beverages they have consumed in the previous 24 h from different perspectives. Interviewers also used food model booklets to further assist participants describe their consumption. For anthropometry data collection, participants were encouraged to remove shoes and heavy clothing before taking weight and height measurements. Interviewers used digital scales to measure weight (maximum 150 kg) and a stadiometer to measure height (maximum 210 cm). The NHMS was conducted from March 2011 to September 2012. The biomedical data collection involved blood and urine samples collected at collection clinics or via home visits using standard procedures [20]. 

### 2.4. Variables

All survey questions are described in the AHS User Guide [23].

### 2.5. Dependent Variable

The EOSS variable was created using chronic disease biomarkers (for diabetes, cholesterol, triglycerides, kidney disease, and liver enzymes), measured blood pressure, and self-reported long-term health conditions. Specific criteria and thresholds were used to classify study participants in one of the EOSS categories (0–4) (Table S1). Study participants who did not meet at least one of the EOSS 1 to 4 criteria were classified into the EOSS 0 category. When participants fulfilled more than one of the EOSS 1 to 4 criterion, the highest score was used to classify their final EOSS stage. 

### 2.6. Independent Variable

We created our Mediterranean Diet Score (MDS) variable according to one of the most common methods used to assess adherence to Mediterranean diet pattern [14,24]. The methodology used herein has been previously published in detail elsewhere [25]. Briefly, to create the MDS variable, we first calculated sex-specific medians for each of the nine components of MDS for the 5055 study participants. Second, we assigned a value of 0 or 1 to each observation i.e., for healthy components a consumption equal to or above the median got a value of 1 and otherwise 0 and for unhealthy components, a consumption below the median got a value of 1 otherwise 0. Lastly, the values of each component were summed to create final MDS scores which ranged from 0 to 9. The methodology used herein has been published in detail elsewhere [26]. In the present study, only dietary data from day one of the 24-h recalls was used.

### 2.7. Covariates

We considered the following range of demographic and behavioural covariates in our statistical analyses. Demographic variables included sex, age, country of birth, marital status, highest year of school (determined by asking participants “What is the highest year of primary or secondary school completed?”), Socio-Economic Indexes For Areas (SEIFA) (a measure of socio-economic advantage and disadvantage created from other variables on income, housing, and education according to the residential postcodes of participants) [27], and hours usually worked each week (determined by asking participants “How many hours usually do you work each week?”). Behavioural variables included whether exercise last week met 150 min recommended guidelines according to standard National Physical Activity Guidelines for Australian adults (determined by asking participants questions such as: “In the last week, how many times did you do any other more moderate physical activity that you have not already mentioned? (e.g., gentle swimming, social tennis, golf)” and “In the last week, how many times did you do any vigorous physical activity which made [you/him/her] breathe harder or puff and pant? (e.g., jogging, cycling, aerobics, competitive tennis)”); smoking status, (determined by asking participants questions such as: “Do you currently smoke?” and “Have you ever smoked regularly, that is, at least once a day?”), and dieting, (determined by asking participants if they were currently on any kind of diet to lose weight or for some other health related reason) [20]. 

### 2.8. Sample Size Considerations

Studies with the same sample size as ours (comparing groups of size 2019 and 3036) have 90% power to detect a difference in proportions from 0.50 in group 1 to 0.55 in group 2 and statistically significant at the 0.05 significance level. We conclude that our study is well powered to detect any clinically meaningful differences in EOSS risk category associated with poor diet.

### 2.9. Bias

Out of the 12,366 dwellings in the actual sample, 9519 adequately or fully responded to the first interview of the NNPAS, with an estimated response rate of 77%. As BMI was calculated from objectively measured weight and height, with scales and stadiometers, selection of the overweight sample was error free.

### 2.10. Statistical Analysis

We presented the characteristics of study participants by EOSS categories 0–1 vs. 2–4 as counts and percentages and tested for differences between these two groups using Chi-square tests. We used logistic regression to assess the association between low adherence to MDS (scores 0–4) (unhealthy dietary pattern exposure) vs. high adherence to MDS (scores 5–9) (healthy dietary pattern reference) associated with EOSS 2–4 (dependent variable). We fitted five models to assess the association: unadjusted (Model 1), adjusted for SEIFA, sex, age, country of birth, marital status, hours usually worked each week, and level of highest education (Model 2), adjusted for whether exercise last week met 150 min recommended guidelines and smoking status (Model 3), adjusted for dieting (Model 4), and adjusted for energy (Model 5). 

### 2.11. Sensitivity Analyses

We fitted models using MDS tertiles (Table S2) and quartiles (Table S3) to explore the association between the MDS and EOSS. We also repeated the main analysis using the mean of two dietary recalls using the data from participants who had intakes of both days (*n* = 3438) (Table S4). We further explored the association across age and SEIFA strata and tested for potential confounding. To further determine whether other potential confounders have influenced our result, we used Propensity Score Matching (PSM) to minimize confounding due to selection bias [28]. We used logistic regression to calculate the propensity scores, checked the balance of the scores across low adherence MDS (scores 0–4) and high adherence MDS (scores 5–9), examined the propensity scores graph, checked for covariate balance, and matched the data using the nearest neighbour 1:1 matching with a 0.1 caliper. The PSM excluded observations out of the common support area (Figure S1). We repeated the main analysis on the matched dataset (*n* = 3364) (Table S5). We performed the analyses using IBM SPSS Statistics 25 [29] and ‘Pscore’ and ‘psmatch2′ in STATA 15 [30] to conduct the PSM.

## 3. Results

### 3.1. Descriptive characteristics

The study population included 5055 adults with at least overweight (BMI ≥25 kg/m^2^) of whom 3036 (60.1%) were classified into the EOSS 2–4 group indicating clinically significant weight related complications (Table 1). The percentage distributions for MDS (e.g., poor MDS score 0–4: 67.0% vs. 65.2%) and sex (e.g., male: 51.9% vs. 54.1%) were similar across the two EOSS groups (EOSS 0–1 vs. 2–4) but statistically significantly different for SEIFA (e.g., SEIFA most disadvantaged: 17.4% vs. 21.4%), country of birth (e.g., other countries: 13.4% vs. 15.6%), and marital status (e.g., married/de facto: 53.4% vs. 57.1%).

### 3.2. Main Results

There were no statistically significant associations between adherence to the MDS and EOSS category in the unadjusted analysis in Model 1 or after adjustment for all other covariates in separate Models 2 to 5 (Table 2).

### 3.3. Sensitivity Analyses

We conducted sensitivity analyses to further explore the association between poor diet measured by the adherence to MDS and weight related complications measured by EOSS. After using tertiles (Table S2) and quartiles (Table S3) of the MDS, there was a reverse association in unadjusted models but after controlling for the covariates the significance was lost. Also, the results were not changed after the main analyses were repeated using mean dietary intake of two days (Table S4). Furthermore, the null association between low adherence MDS of 0–4 and EOSS ≥2 remained unchanged after we repeated the analysis on three age strata (Table 3) and five SEIFA strata (Table 4). Furthermore, the null finding persisted even after we tested for associations in the PSM dataset (*n* = 3364) (Table S5).

## 4. Discussion

To our knowledge, this is the first study to comprehensively examine the association between unhealthy dietary pattern and weight related complications. In this nationally representative subsample of the Australian population with overweight and obesity, we found no evidence of an association between unhealthy dietary pattern (low adherence MDS) and weight related complications (EOSS ≥ 2) in the unadjusted analysis and after controlling for covariates. Moreover, this null finding persisted after sensitivity analyses with mean dietary intake of two 24 h dietary recalls, in stratified models by age and SEIFA categories, and after our application of the PSM technique. Based on the results of our cross-sectional study, unhealthy dietary pattern was not found to be a useful indicator of weight related complications in the overweight Australian population.

Our null association is consistent with another cross-sectional study of the AHS data set which reported no association of the MDS, using the same definition as ours, with the presence of diabetes and cardiovascular disease after controlling for covariates [31] and another study which showed inconclusive results for poor diet (defined by lower diet quality scores) across EOSS stages [18]. In contrast, one study found that adequate fruit and vegetable intake (defined as “men >35 servings per week; women >25 servings per week”) was associated with a decreased risk of developing weight related complications (EOSS 2 or 3) [17]. Thus, the limited body of inconsistent evidence indicates that population-wide screening for unhealthy dietary patterns as a strategy for identifying risk of weight related complications in people with overweight and obesity is not yet warranted.

The null association reported herein was unexpected given that there is strong expert opinion favouring the Mediterranean diet pattern for better cardiovascular health outcomes [32,33] and evidence from systematic reviews of randomized controlled trials showing small but significant reductions in blood pressure [34,35]. Furthermore, the Mediterranean diet pattern has been associated with healthy weight outcomes [14] and cardio-vascular benefit [36] in both the Mediterranean and non-Mediterranean regions. In contrast, a recent systematic review of randomized controlled trials concluded that there is genuine uncertainty regarding the effect of the Mediterranean diet pattern on cardiovascular disease outcomes, as the quality of evidence is low to moderate [37]. Moreover, another systematic review reported the evidence on the Mediterranean diet intervention lacks robustness due to insignificant results for primary outcomes or as significant results were fragile (a small number of events needed to change the result from significant to non-significant) [38]. This conflicting evidence requires further examination and theoretical explanation.

### Limitations

There are several plausible explanations for the apparent lack of consistent findings on unhealthy dietary pattern and having or developing weight related complications. First, several limitations in the accuracy and reliability of dietary assessment methods in research are generally well known [39,40]. For instance, although 24-h recall provides detailed intake data, it is a subjective method of assessing diet prone to recall bias [41,42]. Furthermore, the 24-h recall method is unable to capture the day to day or seasonal variation in food intake and reflects the average usual intake of a group [41,43]. Under-reporting is another common source of error in nutrition surveys, as individuals could change or mistakenly misrepresent their diet pattern [44]. More importantly, studies have shown that participants with overweight or obesity could omit some food groups during diet assessment, especially for total energy intake and/or unhealthy foods [20,45,46]. It is estimated in the NNPAS that low energy reporting was more common among those with obesity compared to normal weight individuals (males: 34% vs. 10%; females 37% vs. 16%) [20]. Second, the effects of unhealthy diet on weight related complications may be too small to detect, especially in populations with excess weight. For instance, one randomized controlled trial showed a very small and clinically meaningless (–1.1 mm Hg) decrease in systolic blood pressure and no change in diastolic blood pressure after six months of a Mediterranean diet intervention compared with usual diet controls [47]. Third, the null association may have been confounded by reverse causation. It is plausible to hypothesize that some individuals may have changed their dietary pattern or reporting thereof after a new diagnosis or medical event at the time of the survey. For instance, cohort studies have shown that higher adherence to the MDS was protective of cardiovascular diseases [48,49] while a cross-sectional study, similar to our finding, reported no association [31]. Furthermore, a study has shown that individuals may change their diet after having affected well-being (e.g., due to increase in weight) or learning a health problem [50]. Finally, the AHS was conducted using a multi-stage sampling and experienced some non-response. We did not use the weights provided by the AHS, so our sample and results may vary slightly from what would have been obtained with a completely random sample from the Australian population. But it is hard to see how this could have led to any systematic bias in the analyses. This potential source of bias is most likely to occur in cross-sectional studies such as ours as dietary pattern and weight related complications were measured at one point in time.

## 5. Conclusions

We found no evidence to support the hypothesis that unhealthy dietary pattern (defined by low adherence to the Mediterranean diet pattern), was associated with an increased likelihood of having weight related complications among individuals with overweight and obesity in Australia. Although the null association reported herein is consistent with a growing body of conflicting evidence on the health benefits of the Mediterranean diet, future studies should seek to improve the accuracy and frequency of dietary assessments to reduce several plausible sources of bias.

## Figures and Tables

**Table 1:** Participant characteristics by EOSS category in the AHS 2011 to 2012 (*n* = 5055).

		EOSS Categories		*p*-Value
		Number (percent)		
		EOSS 0–1 *n* = 2019	EOSS 2–4 *n* = 3036	
MDS ^1^	0–4	1353 (67.0)	1980 (65.2)	0.193
	5–9	666 (33.0)	1056 (34.8)	
SEIFA ^2^	Most disadvantaged	352 (17.4)	649 (21.4)	<0.001
	Second quintile	403 (20.0)	693 (22.8)	
	Third quintile	418 (20.7)	581 (19.1)	
	Fourth quintile	394 (19.5)	495 (16.3)	
	Least disadvantaged	452 (22.4)	618 (20.4)	
Sex	Male	1048 (51.9)	1641 (54.1)	0.135
	Female	971 (48.1)	1395 (45.9)	
Country of birth	Australia	1519 (75.2)	2140 (70.5)	0.001
	Other English-speaking countries	230 (11.4)	423 (13.9)	
	Other countries	270 (13.4)	473 (15.6)	
Marital status	Married/de facto	1079 (53.4)	1733 (57.1)	0.011
	Not married	940 (46.6)	1303 (42.9)	
Highest year of school completed	Year 12 or equivalent	1170 (57.9)	1218 (40.1)	<0.001
	Year 11 or equivalent	230 (11.4)	323 (10.6)	
	Year 10 or equivalent	449 (22.2)	831 (27.4)	
	Year 9 or equivalent	96 (4.8)	308 (10.1)	
	Year 8 or below	74 (3.7)	356 (11.7)	
Age	18–34 years	730 (36.2)	270 (8.9)	<0.001
	35–54 years	930 (46.1)	1001 (33.0)	
	≥55 years	359 (17.8)	1765 (58.1)	
Hours usually worked each week	Not in workforce/unemployed	426 (21.1)	1357 (44.7)	<0.001
	1–24 h	253 (12.5)	305 (10.0)	
	25–39 h	477 (23.6)	523 (17.2)	
	40 h and more	863 (42.7)	851 (28.0)	
Whether exercise last week, met 150 min recommended guidelines	Met recommended guidelines	1081 (53.5)	1348 (44.4)	<0.001
	Did not meet or don’t know	938 (46.5)	1688 (55.6)	
Smoking status	Current smoker	424 (21.0)	495 (16.3)	<0.001
	Ex-smoker	620 (30.7)	1,221 (40.2)	
	Never smoked	975 (48.3)	1320 (43.5)	
Dieting	On diet to lose weight	225 (11.1)	193 (6.4)	<0.001
	On diet for health reasons	50 (2.5)	184 (6.1)	
	On diet for both reasons	69 (3.4)	153 (5.0)	
	Not currently on a diet	1675 (83.0)	2506 (82.5)	
Energy mean (SD)		8793.21 (3637.32)	8398.91 (3408.24)	<0.001

^1^ Mediterranean diet score; ^2^ Index of Relative Socio-Economic Disadvantage—2011—Quintiles—National; SD, Standard deviation; Energy (kilojoules).

**Table 2:** Unadjusted and multivariable adjusted associations between low adherence MDS (scores 0–4 vs. 5–9) and weight related complications (EOSS 2–4 vs. 0–1) in the AHS 2011 to 2012 (*n* = 5055).

	Model 1	*P*-Value	Model 2	*p*-Value	Model 3	*p*-Value	Model 4	*p*-Value	Model 5	*p*-Value
MDS	OR (95%CI)		OR (95%CI)		OR (95%CI)		OR (95%CI)		OR (95%CI)	
0–4	0.92 (0.82, 1.04)	0.19	0.96 (0.84, 1.10)	0.55	0.95 (0.83, 1.09)	0.44	0.99 (0.85, 1.12)	0.76	0.98 (0.85, 1.12)	0.74
5–9	Reference		Reference		Reference		Reference		Reference	

Notes: MDS, Mediterranean Diet Score 0–4 (low adherence, exposure) vs. 5–9 (high adherence, healthy pattern reference), Model 1, unadjusted; Model 2, adjusted for SEIFA, sex, age, country of birth, marital status, hours usually worked each week, and level of highest education; Model 3, adjusted for whether exercise last week met 150 min recommended guidelines and smoking status; Model 4, adjusted for dieting; Model 5, adjusted for energy.

**Table 3:** Multivariable adjusted associations between low adherence MDS (scores 0–4 vs. 5–9) and weight related complications (EOSS 2–4 vs. 0–1) by age strata in the AHS 2011 to 2012 (*n* = 5055).

	Age (18–34) (*n* = 1000)	*p*-Value	Age (35–54) (*n* = 1931)	*p*-Value	Age (≥55) (*n* = 2124)	*p*-Value
MDS	OR (95%CI)		OR (95%CI)		OR (95%CI)	
0–4	0.88 (0.64, 1.20)	0.40	1.01 (0.83, 1.23)	0.94	0.93 (0.72, 1.18)	0.50
5–9	Reference		Reference		Reference	

Notes: MDS, Mediterranean Diet Score 0–4 (low adherence, exposure) vs. 5–9 (high adherence, healthy pattern reference), the model is adjusted for SEIFA, sex, country of birth, marital status, hours usually worked each week, level of highest education, whether exercise last week met 150 min recommended guidelines, smoking status, dieting, and energy.

**Table 4:** Multivariable adjusted associations between low adherence MDS (scores 0–4 vs. 5–9) and weight related complications (EOSS 2–4 vs. 0–1) by SEIFA strata in the AHS 2011 to 2012 (*n* = 5055).

	Most Disadvantaged (*n* = 1001)	*p*-Value	Second Quintile (*n* = 1096)	*p*-Value	Third Quintile (*n* = 999)	*p*-Value	Fourth Quintile (*n* = 889)	*p*-Value	Least Disadvantaged (*n* = 1070)	*p*-Value
MDS	OR (95%CI)		OR (95%CI)		OR (95%CI)		OR (95%CI)		OR (95%CI)	
0–4	0.88(0.64, 1.22)	0.43	0.87(0.63, 1.20)	0.41	1.15(0.84, 1.56)	0.38	0.81(0.59, 1.12)	0.20	1.13(0.84, 1.51)	0.43
5–9	Reference		Reference		Reference		Reference		Reference	

Notes: MDS, Mediterranean Diet Score 0–4 (low adherence, exposure) vs. 5–9 (high adherence healthy pattern reference), the model is adjusted for sex, age, country of birth, marital status, hours usually worked each week, level of highest education, whether exercise last week met 150 min recommended guidelines, smoking status, dieting, and energy.

## Data Availability

Publicly available datasets were analyzed in this study. This data can be found here subject to the ABS requirements- https://www.abs.gov.au/websitedbs/D3310114.nsf/home/MicrodataDownload, accessed on 23 August 2019.

## Supplementary Materials

The following are available online at https://www.mdpi.com/article/10.3390/nu13113905/s1, Figure S1. Propensity scores across the Mediterranean diet before and after matching, Table S1. Specific criteria and thresholds for variables used to classify study participants in one of the EOSS categories (0–4), Table S2. Unadjusted and multivariable adjusted associations between low adherence MDS (scores lowest tertile vs. highest tertile) and weight related complications (EOSS 2–4 vs. 0–1) category in the AHS 2011 to 2012 (*n* = 5055), Table S3. Unadjusted and multivariable adjusted associations between low adherence MDS (scores lowest quintile vs. highest quintile) and weight related complications (EOSS 2–4 vs. 0–1) category in the AHS 2011 to 2012 (*n* = 5055), Table S4. Unadjusted and multivariable adjusted associations between low adherence MDS (scores 0–4 vs. 5–9) and weight related complications (EOSS 2–4 vs. 0–1) category in the AHS 2011 to 2012 (*n* = 3438), Table S5. Unadjusted and multivariable adjusted associations between low adherence MDS (scores 0–4 vs. 5–9) and weight related complications (EOSS 2–4 vs. 0–1) category in the AHS 2011 to 2012 (*n* = 3364) (matched data).
