# Supplementary material for: Is the Mediterranean Diet Pattern Associated with Weight Related Health Complications in Adults? A Cross-Sectional Study of Australian Health Survey
Source: Nutrients. 2021 Oct 30;13(11):3905. doi: 10.3390/nu13113905 (PMC8624026; doi:10.3390/nu13113905)
Supplement: Supplementary file 1 [file nutrients-13-03905-s001.zip › Table S1.pdf]

**Table S1.** Specific criteria and thresholds for variables used to classify study participants in one of the EOSS categories (0-4)

| | EOSS categories and criteria | | | | |
|---|---|---|---|---|---|
| **Criteria** | **0** | **1** | **2** | **3** | **4** |
| Fasting plasma glucose (FPG) diabetes status (mmol/L) | | | | | |
| <6.1 | x | | | | |
| 6.1 - <7.0 | | x | | | |
| ≥7.0 | | | x | | |
| HbA1c diabetes status (%) | | | | | |
| <6.0 | x | | | | |
| 6.0 - <6.5 | | x | | | |
| ≥6.5 | | | x | | |
| Systolic Blood Pressure (mmHg) | | | | | |
| <130 | x | | | | |
| ≥130 - 139.9 | | x | | | |
| ≥140 | | | x | | |
| Diastolic Blood Pressure (mmHg) | | | | | |
| <85 | x | | | | |
| ≥85 - 89.9 | | x | | | |
| ≥90 | | | x | | |
| Total Cholesterol (mmol/L) | | | | | |
| <5.0 | x | | | | |
| 5.0 - <6.0 | | x | | | |
| ≥6.0 | | | x | | |
| Fasting triglycerides (mmol/L) | | | | | |
| <1.5 | x | | | | |
| 1.5 - <2.5 | | x | | | |
| ≥2.5 | | | x | | |
| Fasting LDL cholesterol - ranged (mmol/L) | | | | | |
| <2.5 | x | | | | |
| 2.5 - <4.0 | | x | | | |
| ≥4.0 | | | x | | |
| HDL cholesterol - ranged (mmol/L) | | | | | |
| ≥1.5 | x | | | | |
| 1.0 - <1.5 | | x | | | |
| <1.0 | | | x | | |
| Alanine aminotransferase (ALT) - categories (U/L) MALES | | | | | |
| <30 | x | | | | |
| 30 - <40 | | x | | | |
| ≥40 | | | x | | |
| Alanine aminotransferase (ALT) - categories (U/L) FEMALES | | | | | |
| <25 | x | | | | |
| 25 - <35 | | x | | | |
| ≥35 | | | x | | |
| Gamma glutamyl transferase (GGT) - ranged (U/L) MALES | | | | | |
| <30 | x | | | | |

| | | | | | |
|---|---|---|---|---|---|
| 30 - <50 | | x | | | |
| ≥50 | | | x | | |
| Gamma glutamyl transferase (GGT) - ranged (U/L) FEMALES | | | | | |
| <20 | x | | | | |
| 20 - <35 | | x | | | |
| ≥35 | | | x | | |
| Chronic Kidney Disease (CKD) stages | | | | | |
| No indicators of CKD | x | | | | |
| Stage 1 | | x | | | |
| Stage 2 | | | x | | |
| Stage 3a + 3b | | | | x | |
| Stages 4 - 5 | | | | | x |
| Self-reported obesity-related chronic diseases | | | x | | |
| Diabetes mellitus - Type 2 | | | x | | |
| High sugar levels in blood/urine | | | x | | |
| High cholesterol | | | x | | |
| Angina | | x | | | |
| Oedema | | | x | | |
| Other Ischaemic heart diseases | | | | x | |
| Heart failure | | | | x | |
| Hypertensive disease | | | x | | |
| Other cerebrovascular diseases | | | x | | |
| Kidney disease | | | x | | |
| Self-assessed health | | | | | |
| 1. Excellent | x | | | | |
| 2. Very good | x | | | | |
| 3. Good | x | | | | |
| 4. Fair | | x | | | |
| 5. Poor | | | x | | |
