# Supplementary material for: Is the Mediterranean Diet Pattern Associated with Weight Related Health Complications in Adults? A Cross-Sectional Study of Australian Health Survey
Source: Nutrients. 2021 Oct 30;13(11):3905. doi: 10.3390/nu13113905 (PMC8624026; doi:10.3390/nu13113905)
Supplement: Supplementary file 1 [file nutrients-13-03905-s001.zip › Table S2.pdf]

Table S2. Unadjusted and multivariable adjusted associations between low adherence MDS (scores lowest tertile vs highest tertile) and weight related complications (EOSS 2-4 vs 0-1) category in the AHS 2011 to 2012 (n=5,055)

MDS	Model 1 OR (95%CI)	P-value	Model 2 OR (95%CI)	P-value	Model 3 OR (95%CI)	P-value	Model 4 OR (95%CI)	P-value	Model 5 OR (95%CI)	P-value
Lowest adherence	0.83 (0.69,0.99)	0.04	0.88 (0.71,1.08)	0.23	0.86 (0.7,1.07)	0.17	0.9 (0.73,1.11)	0.32	0.89 (0.72,1.11)	0.31
Second tertile	0.85 (0.72,1.02)	0.08	0.87 (0.71,1.06)	0.17	0.86 (0.7,1.05)	0.13	0.86 (0.7,1.05)	0.14	0.86 (0.7,1.05)	0.13
Highest adherence	Reference		Reference		Reference		Reference		Reference	

Notes: Model 1, unadjusted; Model 2, adjusted for SEIFA, sex, age, country of birth, marital status, hours usually worked each week, and level of highest education; Model 3, adjusted for whether exercise last week met 150 minutes recommended guidelines and smoking status; Model 4, adjusted for dieting; Model 5, adjusted for energy
