# Supplementary material for: Is the Mediterranean Diet Pattern Associated with Weight Related Health Complications in Adults? A Cross-Sectional Study of Australian Health Survey
Source: Nutrients. 2021 Oct 30;13(11):3905. doi: 10.3390/nu13113905 (PMC8624026; doi:10.3390/nu13113905)
Supplement: Supplementary file 1 [file nutrients-13-03905-s001.zip › Table S4.pdf]

Table S4. Unadjusted and multivariable adjusted associations between low adherence MDS (scores 0-4 vs 5-9) and weight related complications (EOSS 2-4 vs 0-1) category in the AHS 2011 to 2012 (n=3,438)

	Model 1		Model 2		Model 3		Model 4		Model 5	
MDS	OR (95%CI)	P-value	OR (95%CI)	P-value	OR (95%CI)	P-value	OR (95%CI)	P-value	OR (95%CI)	P-value
0-4	1.00(0.87,1.16)	0.95	0.99(0.84,1.17)	0.91	0.97(0.82,1.15)	0.70	1.00(0.84,1.19)	0.97	1.00(0.84,1.19)	0.98
5-9	Reference		Reference		Reference		Reference		Reference	

Notes: Model 1, unadjusted; Model 2, adjusted for SEIFA, sex, age, country of birth, marital status, hours usually worked each week, and level of highest education; Model 3, adjusted for whether exercise last week met 150 minutes recommended guidelines and smoking status; Model 4, adjusted for dieting; Model 5, adjusted for energy
