# Supplementary material for: Is the Mediterranean Diet Pattern Associated with Weight Related Health Complications in Adults? A Cross-Sectional Study of Australian Health Survey
Source: Nutrients. 2021 Oct 30;13(11):3905. doi: 10.3390/nu13113905 (PMC8624026; doi:10.3390/nu13113905)
Supplement: Supplementary file 1 [file nutrients-13-03905-s001.zip › Table S5.pdf]

Table S5. Unadjusted and multivariable adjusted associations between low adherence MDS (scores 0-4 vs 5-9) and weight related complications (EOSS 2-4 vs 0-1) category in the AHS 2011 to 2012 (n=3,364) (matched data)

	**Model 1**	**P-value**	**Model 2**	**P-value**	**Model 3**	**P-value**	**Model 4**	**P-value**
**MDS**	**OR (95%CI)**		**OR (95%CI)**		**OR (95%CI)**		**OR (95%CI)**	
0-4	0.94(0.81, 1.07)	0.34	0.94(0.80, 1.09)	0.40	0.94(0.80, 1.09)	0.41	0.96(0.82, 1.13)	0.64
5-9	Reference		Reference		Reference			

Notes: MDS, Mediterranean Diet Score 0-4 (low adherence, exposure) and vs 5-9 (high adherence healthy pattern reference), Model 1, unadjusted; Model 2, adjusted for socioeconomic disadvantage, sex, age, country of birth, marital status, hours usually worked each week, and level of highest education; Model 3, adjusted for whether exercise last week met 150 minutes recommended guidelines and smoking status; Model 4, adjusted for dieting
