# Supplementary figures and images for: Is the Mediterranean Diet Pattern Associated with Weight Related Health Complications in Adults? A Cross-Sectional Study of Australian Health Survey
Source: Nutrients. 2021 Oct 30;13(11):3905. doi: 10.3390/nu13113905 (PMC8624026; doi:10.3390/nu13113905)

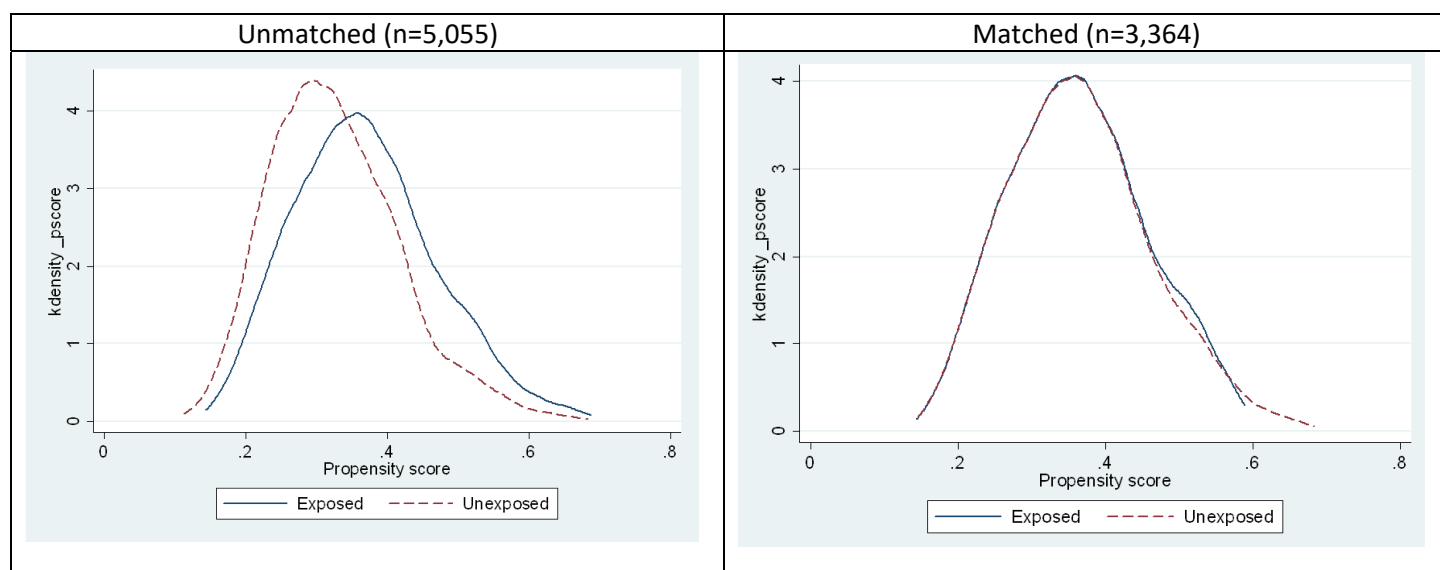

Figure S1: Propensity scores across the Mediterranean diet before and after matching

Supplement: Supplementary file 1 [file nutrients-13-03905-s001.zip › Figure S1.pdf]
